# Sex Differences in the Association of Urinary Concentrations of Phthalates Metabolites with Self-Reported Diabetes and Cardiovascular Diseases in Shanghai Adults

**DOI:** 10.3390/ijerph14060598

**Published:** 2017-06-05

**Authors:** Ruihua Dong, Shanzhen Zhao, Han Zhang, Jingsi Chen, Meiru Zhang, Min Wang, Min Wu, Shuguang Li, Bo Chen

**Affiliations:** 1 Key Laboratory of Public Health Safety of Ministry of Education, Collaborative Innovation Center of Social Risks Governance in Health, School of Public Health, Fudan University, Shanghai 200000, China; 15111020019@fudan.edu.cn (R.D.); 13211020025@fudan.edu.cn (H.Z.); 15211020022@fudan.edu.cn (J.C.); 14211020025@fudan.edu.cn (M.Z.); wumin@shmu.edu.cn (M.W.); 2 Shanghai Entry-Exit Inspection and Quarantine Bureau, Shanghai 200000, China; zhaosz@shciq.gov.cn (S.Z.); wangm@shciq.gov.cn (M.W.)

**Keywords:** phthalates, diabetes mellitus, cardiovascular disease, sex difference, oxidative metabolites, bis (2-ethylhexyl) phthalate

## Abstract

Phthalate exposure was reported to be associated with diabetes mellitus (DM) and cardiovascular disease (CVD). Yet, reported associations and the potential sex differences are inconsistent. We conducted a cross-sectional study involving 2330 participants in the Fall of 2012. Urinary metabolites of 10 phthalates were measured. The status of having DM and CVD-related outcomes were self-reported. In the overall study population, the logistic regression analyses showed that the urinary levels of mono-2-ethyl-5-oxohexyphthalate (MEOHP), mono-2-ethyl-5-hydroxyhexylphthalate(MEHHP) and mono-2-ethyl-5-carboxypentylphthalate (MECPP) were positively associated with DM. Higher urinary levels of monomethyl phthalate (MMP) and mono-2-carboxymethyl-hexyl phthalate (MCMHP) were associated with increased odds of hyperlipidemia, while mono-2-ethylhexylphthalate (MEHP) was significantly inverse-associated with hyperlipidemia. We did not observe significant associations for other CVD-related outcomes with phthalate metabolites. When stratifying by sex, MEHHP, MEOHP, MECPP, MCMHP and the micromolar sums of the oxidative metabolites of DEHP (ΣDEHP_ox_) were all significantly related to DM in males, but not in females. No significant sex differences were found in CVD-related outcomes, except the sporadic associations between phthalates and hyperlipidemia. These findings highlight the importance of investigating the sex-specific relationship between phthalates exposure and DM.

## 1. Introduction

Phthalates, the diesters of 1,2-benzenedicarboxylic acid, are a group of man-made compounds with a various array of uses. They are found in many consumer and industrial products, such as automotive plastics, cosmetics, personal care products, cleaning products, building materials and food packaging [1,2]. Phthalates can be divided into low molecular weight (LMW) and high molecular weight (HMW). High-molecular-weight (HMW) phthalates (>250 Da), such as butyl benzyl phthalate (BBzP), bis(2-ethylhexyl) phthalate (DEHP), di-iso-nonyl phthalate (DiNP), and di-iso-decyl phthalate (DiDP), are mostly used in the production of flexible vinyl plastics, flooring, and medical devices [2,3]. Low-molecular-weight (LMW) phthalates (<250 Da), such as dimethyl phthalate (DMP), diethyl phthalate (DEP), di-iso-butyl phthalate (D_i_BP), and di-n-butyl phthalate (D_n_BP), are commonly used in the production of varnishes, paints, lacquers, and personal care products [4,5]. Because they are not covalently bound to polyvinyl chloride (PVC) in the formulations, phthalates can easily leach out of the plastic products and cause wide contamination in the environment [3,4]. Thus, populations increasingly face the risk of exposure to phthalates through contaminated foods and the environment [5,6].

In recent years, accumulating evidence suggests links between phthalates and diabetes mellitus (DM) and cardiovascular disease (CVD) [7,8]. It has been known for some time that phthalates [9] are PPAR-gamma agonists; they can bind to peroxisome proliferator and activate receptor-alpha and gamma (PPARα and PPARγ), which control carbohydrate metabolism and adipogenesis [10,11,12]. Mechanistically, the close interactions between phthalates and these receptors may impair both beta-cell function and glucose metabolism [13,14,15], and mediate lipid metabolism, leading to an increased risk of DM or hyperlipidemia [16,17,18,19]. Phthalates are known to modulate hormones and inflammatory pathways [20], which could subsequently lead to increased inflammatory profile and insulin resistance [21]. Both adipogenic and inflammatory pathways are known to be involved in hypertriglyceridemia, hyperlipidemia, insulin resistance and hypertension [22]. Therefore, higher phthalate exposure could affect these pathways to alter the risk of DM and CVD. However, the evidence on the association of phthalates with DM and CVD is still limited and inconsistent.

A few cross-sectional studies have revealed a number of interesting relationships between phthalate exposure and insulin resistance and type 2 diabetes [16,17,18,19,23,24,25]. Some studies reported differences in the association of phthalate metabolite concentrations with DM markers by sex but no evident pattern was observed [26,27]. Previous studies have recommended some explanations of such a sex difference, and one possible explanation may be attributed to endogenous hormones. Individual phthalate metabolites have different hormonal and biologic effects. The anti-androgenic effect of the phthalate metabolites may differ according to the levels of endogenous hormones, which vary dramatically by sex [28,29,30]. Therefore, examining the sex difference could lead to a better understanding of the fundamental mechanisms of how phthalate exposure may contribute to sex differences in DM risk.

Regarding CVD, although reports show a potential link to phthalate exposure [31,32], there is no relevant study concerning sex difference. The majority of previous studies have been limited to children, pregnant women, mothers of children in birth cohorts and so on [31,32]. Even in these specific populations, the link between phthalates and CVD is inconsistent [27,33]. Whether or not phthalate exposure contributes to CVD in humans is largely unknown and needs to be further investigated.

In order to better understand the etiology of phthalates’ contribution to DM and CVD, we conducted an exploratory analysis using data from the 2012 Shanghai Food Consumption Survey (SHFCS) and evaluated the associations between 10 phthalate metabolites and self-reported DM and CVD in Shanghai adults.

## 2. Materials and Methods

### 2.1. Study Population and Sampling

The study participants were Shanghai residents who participated in the SHFCS, which was performed by the Fudan University from September 2012 to August 2014 using a four-time 24-h dietary recall questionnaire to collect the seasonal data of food consumption in the community-based general population (Fall 2012, Spring and Winter 2013 and Summer 2014). The community-based SHFCS used the four-stage cluster random sampling method to draw samples [34]. The first stage randomly selected 9 of the 18 districts/counties in Shanghai including Hongkou, Jinshan, Pudong, Qingpu, Baoshan, Huangpu, Xuhui, Putuo and Chongming. The second stage randomly selected 1 to 6 residential communities from each district/county based on the population density. The third stage randomly selected 2 to 4 streets/villages from each community based on the population density. The fourth stage randomly selected 20 households. Finally, 4623 participants from 1760 households were invited to participate the investigation, and 3322 participants from 1325 households finished the investigation.

At the first of the four-time interviews (Fall 2012), urine samples of random spot were obtained from 3082 participants and stored at −20 °C. The urine collection was done for each participant right after they finished the 24-h dietary recall questionnaire and social-demographic questionnaire. The measures of height and weight were also conducted for each participant. Some participants were excluded from the study: 88 for lack of data on the status of CVD; 89 for lack of weight or height information; 326 without enough volume of the urine sample for detecting phthalate metabolites; 25 for unreasonable creatinine concentration (<20 μmol or >30,000 μmol); and 224 aged ≤18 years. Therefore, 2330 participants aged >18 years had complete information and phthalate metabolites.

### 2.2. Identification of Diabetes (DM) and Cardiovascular Diseases (CVD)

The identifications of DM and CVD in this study were according to the self-reported answer to the question: “Have you ever been diagnosed with DM, hypertension, hyperlipidemia, coronary heart disease (CHD) or stroke by a doctor or a physical examination?” Participants defined having those self-reported disease were those who gave the “yes” answer. Participants having CVD were defined as those who have any one of the CHD or stroke. Hypertension and hyperlipidemia were not categorized into the CVD category but treated as CVD risk factors. Both CVD (CHD or stroke) or CVD risk factors (hypertension or hyperlipidemia) were defined as CVD-related outcomes in this study.

### 2.3. Assessment of Phthalate Metabolites in Urine

One spot urine sample from each participant was collected in glass tubes capped with polypropylene lids. Both tubes and lids had been previously washed to remove the background phthalates. Ten phthalate metabolites were measured in this study, including monomethyl phthalate (MMP), monoethylphthalate (MEP), mono-n-butylphthalate (M_n_BP), monoisobutylphthalate (M_i_BP), mono-benzylphthalate (MB_z_P), mono-2-ethylhexylphthalate (MEHP), mono-2-ethyl-5-oxohexyphthalate (MEOHP), mono-2-ethyl-5-hydroxyhexylphthalate (MEHHP), mono-2-ethyl-5-carboxypentylphthalate (MECPP) and mono-2-carboxymethyl-hexyl phthalate (MCMHP). Phthalate metabolites in urine were analyzed by liquid chromatography tandem mass spectrometry (API 4000, LC-MS/MS, Shimadzu, Japan) according to Tranfo et al. [30]. The method has been described in detail in our previous report [35]. Briefly, 1 mL of urine sample was incubated with β-glucuronidase at 37°C for 120 min. The sample was subsequently acidified with 1 mL of aqueous 2% (*v*/*v*) acetic acid, mixed with 100 μL of internal standard (100 μg/L), and loaded into a PLS column previously activated with 2 mL methanol and 2 mL of aqueous 0.5% (*v*/*v*) acetic acid. After sample loading, the column was washed with eluted with 1 mL of methanol and 2 mL of aqueous 0.5% (*v*/*v*) acetic acid. The eluate was passed through a 0.2-μm filter and analyzed (10 μL) by LC-MS/MS coupled to an AQUASIL C18 column.

For the quality control of laboratory procedures, we processed four matrix-spiked samples at two different spiking concentrations (10 and 25 ng/mL) and two procedural blanks in each batch of 30 samples. The average recoveries and relative standard deviations (RSD) of target metabolites in spiked samples ranged from 71.5% to 109.1% and from 1.2% to 7.4% at 10 ng/mL respectively, and ranged from 58.5% to 139.2% and from 0.8% to 8.1% at 25 ng/mL. Sample concentrations of the metabolites with trace blanks were determined after subtraction of the blank values. The method had limits of detection (LOD) of 0.02, 0.20, 0.04, 0.04, 0.20, 0.60, 0.10, 0.20, 0.03, and 0.50 μg/L for MMP, MEP, M_n_BP, M_i_BP, MB_z_P, MEHP, MEOHP, MECPP, MEHHP, and MCMHP, respectively [34].

Additionally, individuals may be exposed to phthalate mixtures at the same time. In order to assess the co-exposure, we performed principal component analysis (PCA) using the measured values of ten metabolites (Table S1, see the Supplementary Materials). The major component accounted for 38.9% of the source variance and was dominated by the 5 metabolites of DEHP. We therefore assessed the co-exposure by calculating the micromolar sum of DEHP metabolites (ΣDEHP) including MEHP, MEHHP, MECPP, MEOHP and MCMHP. In our preliminary analysis, the significant and consistent associations were only observed in the oxidative monoesters of bis (2-ethylhexyl) phthalate (DEHP) including MEOHP, MEHHP, MECPP and MCMHP. When stratifying by sex, we therefore calculated the micromolar sum of these four oxidative metabolites of DEHP (ΣDEHP_ox_) as an indicator.

The concentrations of 10 phthalate metabolites and the micromolar sum were adjusted using creatinine to correct for urine dilution. Urinary creatinine concentrations were analyzed with an enzymatic method on an Architect C8000 automatic biochemical analyzer (ARCHITECT C8000, Abbott Laboratories, Chicago, IL, USA).

### 2.4. Statistical Analysis

The analysis was performed using SPSS version 21.0 Software (SPSS, Inc., Chicago, IL, USA). We used a complex sampling design of taking a four-stage method. In order to prevent bias, the analysis of survey data from complex design requires software that can incorporate the sampling weights and survey design. Therefore, complex samples (SPSS 21.0) was used when performing all statistical analyses. The concentrations of phthalate metabolites were corrected for urine dilution by urine creatinine, as recommended by a previous methodology [35]. Urinary concentrations of metabolites below the LOD were assigned a value of 1/2 LOD. Two-sided *p*-values < 0.05 were considered to be statistically significant. We estimated the association between each metabolite of phthalates and the status of DM and CVD-related outcomes in the overall study population, then an additional stratified analysis was conducted to explore the sex differences. Phthalate concentrations were treated as categorical variables (categorized into quartiles) in the logistic regression analyses. In the preliminary analysis in the overall population, we constructed two adjusted models. One was forcing all confounders into the regression models. The potential confounders were age, sex, educational level (less than or equal to primary school, middle school/technical secondary school, and college or greater), marriage (married, other), smoking status (current/past, or never), body mass index (BMI), total caloric intake and total fat intake. Another model was constructed without adjusting the dietary factors (total caloric intake and total fat intake). The odds ratios (ORs) and 95% confidence intervals (CIs) were calculated for each higher quartile (Q) comparing to the lowest quartile. We also calculated the *p*-value for trend (*p* for trend) across the lowest quartile (Q1) to the highest quartile (Q4).

We also conducted sensitivity analyses to assess the robustness of our findings by exploring the associations between phthalates and target outcomes in populations without other self-reported outcomes. For example, when exploring whether self-reported hypertension was or was not associated with phthalate metabolites, the analyses were only restricted to the overall population (or to the sub-population of men or women) without other self-reported outcomes including CVD and DM.

### 2.5. Ethics Approval and Consent to Participate

All subjects submitted written informed consent before their participation in the survey. The study was approved by the local authorities and the Ethics Committee of School of Public Health at Fudan University (IRB#2011-03-0264).

## 3. Results

Table 1 shows the baseline characteristics. The study population consisted of 1108 men (47.6%) and 1222 women (52.4%). Median age is 53 years.

Table 2 presented the percentiles and geometric mean (GM) of each measured metabolite of phthalates. The detection rates of MECPP, MEHHP, MEHP MCMHP and MEOHP were 99.8%, 99.7%, 96.8%, 96.6%, 91.7% respectively. In this study, the median concentrations of MECPP, MEHHP, MCMHP and MEOHP were 13.95, 11.64, 17.56, 4.67 μg/g, respectively. These concentrations were similar to the previous reports in general populations from China and USA [36,37], but lower than that in Mexico [38]. The concentrations of M_i_BP (8.42) and M_n_BP (12.92) in this study was lower than the reported values in previous studies from China [36,39].

Table 3 shows the adjusted odds of each individual outcome (DM vs. normal; hypertension vs. normal; hyperlipidemia vs. normal; CHD vs. normal; stroke vs. normal; CVD vs. normal) by quartile of phthalate concentrations. Since two adjusted models showed similar results, we kept the adjusted results in Table 3, and put the non-adjusted (without adjusting the dietary factors) results in Table S2 (See the Supplementary Materials).

In the overall study population, urinary levels of MEOHP, MEHHP and MECPP showed positive associations with DM. Significantly increased odds (shown as OR (95% CI)) were observed in Q3 of MEOHP (1.76 (1.07, 2.91)); Q2 (1.72 (1.01, 2.92)) and Q3 (1.92 (1.14, 3.25)) of MEHHP; Q3 (1.74 (1.04, 2.91)) and Q4 (1.86 (1.11, 3.14)) of MECPP. The *p*-values for the trend (*p* for trend) were 0.013, 0.044 and 0.010 for MEOHP, MEHHP and MECPP, respectively.

In the overall population, higher urinary levels in Q4 of MMP (2.38 (1.35, 4.17)) and Q3 of MCMHP (1.86 (1.05, 3.30)) were associated with increased odds of hyperlipidemia. Both MMP and MCMHP had significant *p* for trend (MMP: 0.002; MCMHP: 0.032) in their associations with hyperlipidemia. While MEHP was significantly inverse-associated with hyperlipidemia in Q3 vs. Q1 (0.49 (0.27, 0.92)), it showed no significant *p* for trend. We did not observe significant associations for other types of CVD-related outcomes with phthalate metabolites.

**Figure 1:**
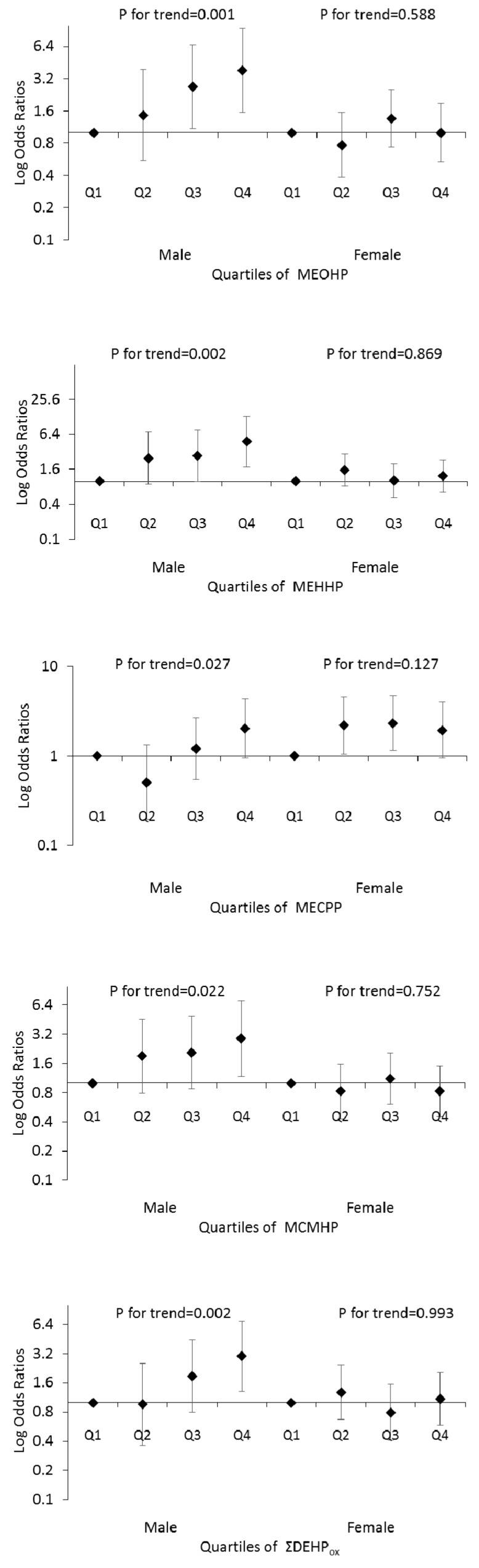
Logistic regression analyses of quartile metabolites (oxidative monoesters and their micromolar sum (ΣDEHP_ox_)) of DEHP in association with self-reported DM. The Log odds ratios and 95% confidence intervals are presented for each higher quartile compared to the lowest quartile (Q1). The models were adjusted for age, marriage, education, smoking status, body mass index, total caloric intake and total fat intake.

When stratifying by sex, phthalate associations with self-reported DM were only found in the oxidative metabolites of DEHP (Figure 1). In males, significant increased ORs were found in the Q3 (2.69 (1.08, 6.65)) and Q4 (3.83 (1.55, 9.44)) of MEOHP, in the Q4 of MEHHP (4.78 (1.77, 12.92)), in the Q4 of MCMHP (2.89 (1.18, 7.09)) and in the Q4 of ΣDEHP_ox_ (2.99 (1.30, 6.86)). No significant ORs were found in the different quartiles of male MECPP compared to Q1. However, the significant *p* for trend in MECPP was also found. The significant *p*s for trend were 0.001, 0.002, 0.027, 0.022 and 0.002 for MEOHP, MEHHP, MECPP, MCMHP and ΣDEHP_ox_, respectively. In contrast to the males, no significant association was found in the females.

When stratifying by sex, phthalate associations with the CVD-related outcomes were only found in the hyperlipidemia. In females, significant increased ORs were found in the Q2 (3.65 (1.41, 9.45)) and Q4 (4.10 (1.60, 10.50)) of M_i_BP, in the Q4 of MEP (2.24 (1.08, 4.63)) and in the Q4 of MMP (2.89 (1.34, 6.23)). The significant *p*s for trend were 0.012, 0.006 and 0.004 for M_i_BP, MEP and MMP, respectively. In males, significant increased ORs were found in the Q4 (2.60 (1.14, 5.95)) of MCMHP (4.78 (1.77, 12.92)). The significant *p* for trend was 0.041. There is no sex difference in the associations between phthalates and other CVD-related outcomes (data are not shown).

## 4. Discussion

In this study, we found that the increased odds of having self-reported DM were associated with higher levels of the oxidative metabolites (MEOHP, MEHHP, MECPP and MCMHP) of DEHP, but not the hydrolytic metabolite (MEHP). This finding indicated that the oxidative metabolites of DEHP may serve as better biomarkers for identifying the effective dose–response relationship with regard to phthalates toxicity. There are several reasons that support this point. First, DEHP oxidative metabolites are of higher stability and sensitivity in determination, whereas MEHP has a shorter serum half-life than the other metabolites [40]. The detection rates of four DEHP oxidative metabolites (MEOHP, MECPP, MEHHP, MCMHP) in our samples were similar to the relevant studies published from 2007 to 2016, which were all above 90% [39,41,42,43,44,45]. Second, it has been reported that MEHHP and MEOHP was more active in animals than MEHP [46]. Third, it has been reported that the level of hydrolytic monoesters of phthalates could have been easily contaminated from the environment during the sampling and analyzing process, while the oxidative monoesters are not easy to contaminate [47]. These reasons may explain why the oxidative metabolites of DEHP in our data have greater power in identifying the possible risk relationship with self-reported diseases.

The major finding of our study is the sex difference in the association between phthalates and self-reported DM. Only the males, not the females, were found to be consistently associated with the oxidative metabolites of DEHP. To our knowledge, previous studies have only presented sporadic sex associations with phthalate exposure and found no consistent pattern [26,27].

Previous studies have reported positive associations between higher levels of certain phthalate metabolites and DM (or insulin resistance) [25,26]. These associations differed between males and females. For males, a male subset of the National Health and Nutrition Examination Survey (NHANES) in 1999–2002 found that MEP and MB_z_P were associated with increases in the homeostasis model assessment estimated insulin resistance (HOMA-IR) [25]. The NHANES 2001–2008 study showed a stronger association between ΣDEHP and fasting glucose in males than females, which was similar to our results [26]. For females, an association between MB_z_P and insulin resistance or a higher prevalence of DM was observed from two NHANES surveys [18,27]. Nevertheless, compared with NHANES data, MB_z_P was inversely associated with DM in Mexican females [19]. In a recent NHANES 2001–2010 study, analyses were stratified by age, sex and menopausal status; independent of age, there was a different association between certain phthalate metabolites and metabolic syndrome (MetS) based on menopausal status; the association between ΣDEHP and MetS was only seen among women younger than 50 years. The population in our study is skewed distribution in age (median age = 53 years; 63% of the females were >50 years old), and most females may already have been in menopause. In females, menopause-induced oestrogen deficiency and increased androgenicity are associated with concomitant alterations in the metabolic risk profile [48]. Therefore, the evident sex difference observed in our population may be attributed to the endogenous sex hormones.

Until recently, increasing evidence supporting the role of endogenous sex hormones in DM development had come from clinical and animal observations [49,50,51]. There were clear sex differences in how sex steroid hormones may modulate the risk of DM [49,50]. Endogenous levels of testosterone and sex-hormone-binding globulin (SHBG) each exhibit sex-dependent relations with a risk of DM [51]. As an endocrine disruptor, phthalates may modulate sex hormone biology through binding sex hormone receptors and interfering in the signalling pathways [49].

Several epidemiological studies have pointed out that increasing exposure to phthalates inhibiting steroidogenesis and reducing serum testosterone levels in both males and females in differing age groups [52,53,54,55]. Lower levels of testosterone were reported to be associated with a lower risk of DM in females, but a higher risk in males [49]. In this pathway of reducing testosterone, phthalates may decrease the risk of DM in females but increase the risk in males, which may explain that the stronger positive associations in our data lie in males but not in females.

SHBG is hormone-related biomarker that has been reported to affect free-circulating hormone levels and represents a potential target for the phthalates endocrine disruptor function in the human body [56]. Phthalates have been suggested to have a potential disrupting activity in the endocrine homeostasis function of SHBG [57,58,59]. The association between low SHBG and the development of DM has been reported in both sex, and the inverse association of SHBG with the risk of DM was stronger in females than in males [50,60]. In addition, multiple studies reported no inverse associations between SHBG and DM in males; that is, SHBG appeared to be more protective in females than in males [61]. Furthermore, one study has indicated that SHBG had a significant inverse association with insulin resistance only in postmenopausal women [48]. Based on the above reports, it is possible to deduce that the effects of phthalates on DM through the SHBG pathway were negative in males, but positive in females. Our data of phthalate-associated DM in males may be mainly associated with testosterone but not SHBG. It is unclear why we did not observe the inverse associations between phthalates and DM in females. Importantly, our observations regarding testosterone and SHBG make it reasonable to deduce that phthalates are the possible risk factors of DM in males, but not in females.

In this study, another interesting observation was that we did not find phthalate associations with CVD-related outcomes in both the overall population and in the separated sex populations. We only found some sporadic associations between phthalates and hyperlipidemia. Previous studies have reported positive associations between phthalates and hypertension, stroke and hypertriglyceridemia. However, this existing evidence is very limited and inconsistent. For example, according to the data of NHANES 2001–2004, urine phthalate concentrations are higher in people with stroke [62]. Phthalates were positively associated with hypertension, but not with triglycerides or high-density lipoproteins in children and adolescents [31,32]. The urine ΣDEHP were associated with hypertriglyceridemia in the overall study population from NHANES 2001–2010 [27], while no significant associations were found in females from NHANES 1999–2004 [33]. The differences between our results and those of other studies could be due to the different population samples and statistical methods, and the self-reported design may impact the accuracy of sample classification. As the lack of existing evidence, other explanations for the associations may be involved.

The present study has some limitations that deserve discussion. One weakness is the cross-sectional design, where phthalate exposure was measured by a one-time collection of urine samples. Human urine can be easily affected by many factors, such as the timing of sampling or other routes of exposure (e.g., personal care products, floor coverings, medications). In this case, the urine data may not be the truly representative of phthalate exposure. It is unclear to what extent that a single measurement from a cross-sectional design may reflect long-term exposure because of the short half-lives of phthalates in urine. There is a view that one spot urine sample was able to reflect long-term exposure to phthalates because study participants may maintain the same lifestyle with unaltered exposure patterns [63]. It is also unclear whether higher levels of phthalate metabolites were the reason or the consequence of studied outcomes. For example, patients with DM may metabolize phthalates differently or may behaviorally operate differently (take more medication use as an example) leading to increased exposure to phthalates. This possibility of reverse causation could disturb the deduction of attributing DM risk to phthalate exposure. Another weakness is that the status of DM and CVD in our data were self-reported which may have introduced the recall bias, leading to the misclassification for some individuals. It is generally the truth that the prevalence of the disease (DM or CVD) was much lower defined by self-reported questionnaire than the medical examination, and the less number of identified cases may lead to the non-significance associations between self-reported disease and phthalate exposure.

## 5. Conclusions

In this study, the association between higher levels of certain phthalates (oxidative metabolites of DEHP) and DM was found in males, but not females. Regardless of sex, we only found some sporadic associations between phthalates and hyperlipidemia. We did not find phthalate associations with other CVD-related outcomes. These findings highlight the importance of investigating the sex-specific relationship between phthalates exposure and DM.

## Figures and Tables

**Table 1:** Demographic characteristics of study population (*n* = 2330).

Characteristic	Category	Total)
Age, median (IQR), year	-	53 (41, 64)
Nationality, *n* (%)	Han	2308 (99.1)
	Others	22 (0.9)
Sex	Male	1108 (47.6)
	Female	1222 (52.4)
Education, *n* (%)	≤Primary school	519 (22.5)
	Middle school/technical, secondary school	1398 (60.5)
	≥College graduate	392 (17.0)
Marriage, *n* (%)	Married	1950 (85.8)
	Others	322 (14.2)
Smoking, *n* (%)	Never smoker	1743 (74.8)
	Current/past smoker	587 (3.9)
Total calories, median (IQR), kcal	-	1631 (1219, 1888)
Total fat, median (IQR), g	-	54.3 (32.9, 69.2)
Height, median (IQR), m	-	1.65 (1.59, 1.70)
Weight, median (IQR), kg	-	64.0 (55.0, 70.0)
BMI, median (IQR), kg/m^2^	-	23.5 (21.3, 25.7)
DM, *n* (%)	no	2150 (92.3)
	yes	180 (7.7)
CVD, *n* (%)	no	2187 (93.9)
	yes	130 (6.1)
Hypertension, *n* (%)	no	1699 (72.9)
	yes	631 (27.1)
Hyperlipidemia, *n* (%)	no	2167 (93.6)
	yes	148 (6.4)
CHD, *n* (%)	no	2203 (95.1)
	yes	114 (4.9)
Stroke, *n* (%)	no	2300 (99.3)
	yes	16 (0.7)

BMI = body mass index; DM = diabetes mellitus; CVD = cardiovascular disease; CHD = coronary heart disease; IQR = interquartile range.

**Table 2:** The distributions of phthalate metabolites after corrected by urine creatinine (*n* = 2330).

	*n* > LOD (%)	Percentiles					
		5th	25th	50th	75th	95th	GM
MMP	2076 (89.1)	0.02	1.01	2.93	6.42	19.91	1.84
MEP	2113 (90.7)	0.20	3.30	8.82	20.80	82.56	7.29
M_n_BP	1731 (74.3)	0.02	0.17	12.92	37.10	111.49	4.01
M_i_BP	1915 (82.2)	0.03	1.91	8.42	19.38	62.77	3.95
MB_z_P	1637 (70.3)	0.21	0.84	2.07	4.10	22.36	1.95
MEHP	2255 (96.8)	0.83	4.42	8.84	18.21	70.95	8.56
MEOHP	2136 (91.7)	0.07	2.66	4.67	8.05	20.57	3.66
MECPP	2325 (99.8)	4.24	8.30	13.95	24.14	68.51	14.71
MEHHP	2323 (99.7)	0.29	4.26	11.64	22.57	70.24	8.11
MCMHP	2250 (96.6)	3.71	10.84	17.56	30.03	79.53	16.91

LOD, limit of detection; GM: geometric mean; MMP: monomethyl phthalate; MEP: monoethylphthalate; MnBP: mono-n-butylphthalate; MiBP: monoisobutylphthalate; MBzP: mono-benzylphthalate; MEHP: mono-2-ethylhexylphthalate; MEOHP: mono-2-ethyl-5-oxohexyphthalate; MEHHP: mono-2-ethyl-5-hydroxyhexylphthalate; MECPP: mono-2-ethyl-5-carboxypentylphthalate; MCMHP: mono-2-carboxymethyl-hexyl phthalate.

**Table 3:** Logistic regression analyses of quartile metabolites of phthalates in association with self-reported DM and CVD-related outcomes (*n* = 2330).

Phthalates Metabolites			DM		Hypertension		Hyperlipidemia		CHD		Stroke		CVD	
		**N**	***n*** ** (%)**	**OR (95% CI) ^a^**	***n*** ** (%)**	**OR (95% CI)**	***n*** ** (%)**	**OR (95% CI)**	***n*** ** (%)**	**OR (95% CI)**	***n*** ** (%)**	**OR (95% CI)**	***n*** ** (%)**	**OR (95% CI)**
MMP	Q1	569	38 (6.7%)	1	142 (25.0%)	1	23 (4.1%)	1	25 (4.4%)	1	2 (0.4%)	1	27 (4.8%)	1
	Q2	582	37 (6.4%)	0.95 (0.57, 1.58)	140 (24.1%)	0.94 (0.68, 1.30)	31 (5.4%)	1.29 (0.69, 2.41)	27 (4.7%)	1.05 (0.57, 1.94)	5 (0.9%)	2.34 (0.44, 12.43)	32 (5.5%)	1.17 (0.66, 2)
	Q3	588	37 (6.3%)	0.90 (0.54, 1.51)	155 (26.4%)	0.98 (0.71, 1.35)	35 (6.0%)	1.34 (0.72, 2.49)	20 (3.4%)	0.69 (0.35, 1.36)	2 (0.3%)	1.09 (0.15, 7.91)	22 (3.8%)	0.96 (0.69, 1.32)
	Q4	584	68 (11.6%)	1.54 (0.96, 2.45)	194 (33.2%)	1.20 (0.87, 1.65)	59 (10.2%)	2.38 (1.35, 4.17) *	42 (7.2%)	1.28 (0.72, 2.28)	7 (1.2%)	4.06 (0.77, 21.40)	49 (8.4%)	1.32 (0.96, 1.81)
	*p* ^b^			0.085		0.254		0.002		0.560		0.209		0.408
MEP	Q1	568	39 (6.9%)	1	150 (26.4%)	1	32 (5.7%)	1	25 (4.4%)	1	0 (0.0%)	1	25 (4.4%)	1
	Q2	583	40 (6.9%)	1.29 (0.79, 2.12)	146 (25.0%)	1.13 (0.82, 1.54)	25 (4.3%)	0.78 (0.43, 1.43)	27 (4.7%)	1.27 (0.69, 2.33)	4 (0.7%)	1.10 (0.72, 1.83)	31 (5.4%)	1.48 (0.83, 2.66)
	Q3	579	39 (6.7%)	1.09 (0.65, 1.83)	155 (26.8%)	1.14 (0.83, 1.56)	35 (6.1%)	0.91 (0.51, 1.63)	23 (4.0%)	0.76 (0.39, 1.50)	5 (0. 9%)	1.04 (0.65, 1.68)	28 (4.9%)	1.00 (0.53, 1.89)
	Q4	593	62 (10.5%)	1.60 (0.99, 2.58)	180 (30.4%)	1.26 (0.92, 1.72)	56 (9.5%)	1.66 (0.99, 2.77)	39 (6.6%)	1.36 (0.76, 2.44)	7 (1.2%)	1.38 (0.87, 2.22)	46 (7.8%)	1.73 (0.98, 3.05)
	*p*			0.013		0.246		0.035		0.538		0.021		0.166
M_i_BP	Q1	583	44 (7.5%)	1	165 (28.3%)	1	27 (4.6%)	1	25 (4.3%)	1	2 (0.3%)	1	27 (4.6%)	1
	Q2	593	46 (7.8%)	1.18 (0.74, 1.91)	157 (26.5%)	0.84 (0.62, 1.15)	43 (7.3%)	1.66 (0.94, 2.93)	35 (5.9%)	1.56 (0.87, 2.81)	5 (0.8%)	2.41 (0.46, 12.73)	40 (6.8%)	1.69 (0.97, 2.94)
	Q3	579	45 (7.8%)	1.32 (0.81, 2.17)	138 (23.8%)	0.80 (0.58, 1.11)	35 (6.1%)	1.33 (0.73, 2.43)	22 (3.8%)	0.85 (0.43, 1.69)	1 (0.2%)	0.60 (0.05, 6.84)	23 (4.0%)	0.80 (0.41, 1.55)
	Q4	568	45 (7.9%)	1.04 (0.63, 1.71)	171 (30.1%)	0.91 (0.66, 1.26)	43 (6.4%)	1.52 (0.85, 2.69)	32 (5.7%)	1.19 (0.64, 2.22)	8 (1.4%)	4.90 (0.95, 25.20)	40 (7.1%)	1.42 (0.79, 2.53)
	*p*			0.786		0.522		0.305		0.965		0.119		0.698
M_n_BP	Q1	581	47 (8.1%)	1	170 (29.3%)	1	28 (4.8%)	1	32 (5.5%)	1	2 (0.3%)	1	34 (5.9%)	1
	Q2	579	34 (5.9%)	0.84 (0.51, 1.38)	147 (25.4%)	0.85 (0.62, 1.16)	38 (6.6%)	1.69 (0.97, 2.95)	20 (3.5%)	0.73 (0.39, 1.36)	3 (0.5%)	1.79 (0.29, 11.13)	23 (4.0%)	0.76 (0.42, 1.36)
	Q3	584	44 (7.5%)	1.19 (0.74, 1.93)	146 (25.0%)	0.80 (0.58, 1.10)	42 (7.3%)	1.31 (0.73, 2.33)	28 (4.8%)	0.82 (0.45, 1.51)	5 (0.9%)	2.97 (0.55, 15.92)	33 (5.7%)	0.95 (0.54, 1.68)
	Q4	579	55 (9.5%)	1.09 (0.68, 1.75)	168 (29.0%)	0.76 (0.55, 1.05)	40 (7.0%)	1.20 (0.67, 2.12)	34 (5.9%)	0.86 (0.48, 1.53)	6 (1.0%)	3.99 (0.71, 22.42)	40 (7.0%)	1.02 (0.59, 1.75)
	*p*			0.469		0.084		0.823		0.711		0.103		0.797
MB_z_P	Q1	557	46 (8.3%)	1	164 (29.4%)	1	34 (6.1%)	1	31 (5.6%)	1	4 (0.7%)	1	35 (6.3%)	1
	Q2	596	34 (5.7%)	0.67 (0.40, 1.13)	140 (23.5%)	0.78 (0.56, 1.08)	30 (5.1%)	0.88 (0.49, 1.58)	20 (3.4%)	0.67 (0.35, 1.29)	7 (1.2%)	2.15 (0.53, 8.63)	27 (4.6%)	0.81 (0.45, 1.47)
	Q3	584	49 (8.4%)	0.93 (0.57, 1.51)	158 (27.1%)	0.76 (0.55, 1.06)	43 (7.4%)	1.03 (0.58, 1.80)	26 (4.5%)	0.71 (0.38, 1.34)	2 (0.3%)	0.61 (0.10, 3.77)	28 (4.8%)	0.72 (0.39, 1.31)
	Q4	586	51 (8.7%)	1.06 (0.66, 1.69)	169 (28.8%)	0.97 (0.71, 1.33)	41 (7.0%)	1.31 (0.77, 2.24)	37 (6.3%)	1.26 (0.72, 2.23)	3 (0.5%)	0.87 (0.17, 4.46)	40 (6.8%)	1.27 (0.74, 2.17)
	*p*			0.491		0.922		0.228		0.284		0.410		0.398
MEHP	Q1	579	44 (7.6%)	1	160 (27.6%)	1	41 (7.1%)	1	30 (5.2%)	1	5 (0.9%)	1	35 (6.1%)	1
	Q2	576	37 (6.4%)	0.95 (0.58, 1.57)	137 (23.8%)	0.92 (0.67, 1.28)	28 (4.9%)	0.68 (0.39, 1.20)	14 (2.4%)	0.47 (0.23, 0.95)	4 (0.7%)	0.86 (0.23, 3.29)	18 (3.1%)	0.51 (0.27, 0.96)
	Q3	591	44 (7.4%)	0.97 (0.59, 1.59)	136 (23.0%)	0.73 (0.53, 1.02)	26 (4.4%)	0.49 (0.27, 0.92) *	28 (4.8%)	0.85 (0.46, 1.55)	3 (0.5%)	0.64 (0.15, 2.81)	31 (5.3%)	0.78 (0.45, 1.37)
	Q4	577	55 (9.5%)	1.10 (0.69, 1.77)	198 (34.3%)	1.16 (0.85, 1.59)	53 (9.3%)	1.15 (0.70, 1.88)	42 (7.3%)	0.99 (0.56, 1.72)	4 (0.7%)	0.66 (0.15, 2.92)	46 (8.0%)	0.90 (0.53, 1.53)
	*p*			0.682		0.609		0.656		0.606		0.385		0.997
MEOHP	Q1	587	35 (6.0%)	1	159 (27.1%)	1	30 (5.1%)	1	28 (4.8%)	1	4 (0.7%)	1	32 (5.5%)	1
	Q2	607	30 (4.9%)	0.96 (0.55, 1.68)	148 (24.4%)	0.88 (0.64, 1.22)	27 (4.5%)	0.74 (0.40, 1.38)	30 (5.0%)	1.16 (0.63, 2.11)	4 (0.7%)	1.06 (0.25, 4.40)	34 (5.6%)	1.10 (0.63, 1.93)
	Q3	596	61 (10.2%)	1.76 (1.07, 2.91) *	150 (25.2%)	0.73 (0.52, 1.01)	47 (8.0%)	1.16 (0.66, 2.03)	25 (4.2%)	0.66 (0.34, 1.26)	3 (0.5%)	0.59 (0.10, 3.35)	28 (4.7%)	0.62 (0.33, 1.14)
	Q4	533	54 (10.1%)	1.62 (0.97, 2.70)	174 (32.6%)	1.09 (0.78, 1.50)	44 (8.3%)	1.52 (0.88, 2.62)	31 (5.9%)	1.03 (0.56, 1.90)	5 (0.9%)	1.47 (0.37, 5.82)	36 (6.8%)	1.01 (0.57, 1.79)
	*p*			0.013		0.881		0.046		0.737		0.865		0.611
MEHHP	Q1	563	29 (5.2%)	1	145 (25.8%)	1	29 (5.2%)	1	29 (5.2%)	1	2 (0.4%)	1	31 (5.5%)	1
	Q2	605	47 (7.8%)	1.72 (1.01, 2.92) *	145 (24.0%)	0.76 (0.54, 1.06)	33 (5.5%)	0.90 (0.50, 1.60)	26 (4.3%)	0.61 (0.33, 1.13)	4 (0.7%)	2.20 (0.38, 12.64)	30 (5.0%)	0.68 (0.39, 1.23)
	Q3	609	50 (8.2%)	1.41 (0.82, 2.44)	163 (26.8%)	0.79 (0.57, 1.10)	53 (8.8%)	1.26 (0.73, 2.19)	30 (5.0%)	0.65 (0.35, 1.19)	6 (1.0%)	2.79 (0.52, 15.10)	36 (6.0%)	0.74 (0.42, 1.31)
	Q4	549	54 (9.9%)	1.92 (1.14, 3.25) *	178 (32.6%)	1.22 (0.88, 1.69)	33 (6.1%)	1.02 (0.57, 1.81)	29 (5.3%)	0.77 (0.42, 1.40)	4 (0.7%)	2.26 (0.40, 12.85)	33 (6.1%)	0.81 (0.46, 1.44)
	*p*			0.044		0.169		0.635		0.531		0.438		0.557
MECPP	Q1	600	28 (4.7%)	1	149 (24.8%)	1	30 (5.0%)	1	26 (4.3%)	1	4 (0.7%)	1	30 (5.0%)	1
	Q2	613	40 (6.5%)	1.29 (0.75, 2.23)	152 (24.8%)	0.90 (0.65, 1.24)	29 (4.8%)	1.04 (0.57, 1.89)	23 (3.8%)	0.79 (0.42, 1.50)	4 (0.7%)	0.86 (0.20, 3.69)	27 (4.4%)	0.79 (0.44, 1.42)
	Q3	595	57 (9.6%)	1.74 (1.04, 2.91) *	160 (26.9%)	0.91 (0.66, 1.25)	48 (8.1%)	1.48 (0.85, 2.59)	33 (5.6%)	1.02 (0.56, 1.85)	4 (0.7%)	0.76 (0.16, 3.52)	37 (6.3%)	0.93 (0.53, 1.63)
	Q4	515	55 (10.7%)	1.86 (1.11, 3.14) *	170 (33.0%)	1.26 (0.91, 1.75)	41 (8.0%)	1.53 (0.87, 2.71)	32 (6.3%)	1.04 (0.56, 1.92)	4 (0.8%)	1.12 (0.26, 4.84)	36 (7.0%)	0.98 (0.55, 1.73)
	*p*			0.010		0.190		0.071		0.650		0.926		0.910
MCMHP	Q1	604	38 (6.3%)	1	159 (26.3%)	1	26 (4.3%)	1	31 (5.2%)	1	4 (0.7%)	1	35 (5.8%)	1
	Q2	602	41 (6.8%)	1.14 (0.68, 1.90)	155 (25.7%)	1.02 (0.74, 1.40)	35 (5.9%)	1.39 (0.76.2.54)	30 (5.0%)	1.01 (0.56, 1.81)	5 (0.8%)	1.34 (0.35, 5.13)	35 (5.8%)	1.06 (0.62, 1.83)
	Q3	599	56 (9.3%)	1.42 (0.87, 2.32)	149 (24.9%)	0.81 (0.58, 1.12)	50 (8.4%)	1.86 (1.05, 3.30) *	22 (3.7%)	0.63 (0.33, 1.18)	3 (0.5%)	0.55 (0.10, 3.07)	25 (4.2%)	0.60 (0.33, 1.09)
	Q4	518	45 (8.7%)	1.26 (0.76, 2.08)	169 (32.4%)	1.22 (0.88, 1.69)	37 (7.2%)	1.78 (0.98, 3.20)	31 (6.0%)	0.98 (0.54, 1.76)	4 (0.8%)	1.12 (0.26, 4.72)	35 (6.8%)	0.98 (0.56, 1.69)
	*p*			0.266		0.514		0.032		0.657		0.676		0.490
ƩDEHP	Q1	556	34 (5.8%)	1	149 (25.3%)	1	31 (5.3%)	1	28 (4.8%)	1	5 (0.8%)	1	33 (5.6%)	1
	Q2	550	37 (6.3%)	1.08 (0.63, 1.84)	133 (22.7%)	0.90 (0.64, 1.25)	32 (5.5%)	1.13 (0.63, 2.04)	23 (4.0%)	0.09 (0.48, 1.69)	5 (0.9%)	1.03 (0.28, 3.78)	28 (4.8%)	0.91 (0.52, 1.62)
	Q3	532	50 (8.6%)	1.06 (0.63, 1.77)	158 (27.1%)	0.81 (0.58, 1.13)	37 (6.4%)	0.87 (0.48, 1.59)	24 (4.1%)	0.59 (0.31, 1.12)	2 (0.3%)	0.19 (0.02, 1.71)	26 (4.5%)	0.51 (0.28, 0.95)
	Q4	505	59 (10.5%)	1.40 (0.85, 2.31)	191 (33.9%)	1.22 (0.88, 1.68)	48 (8.6%)	1.44 (0.83, 2.51)	39 (7.0%)	0.96 (0.53, 1.73)	4 (0.7%)	0.77 (0.19, 3.12)	43 (7.7%)	0.92 (0.53, 1.60)
	*p*			0.195		0.308		0.287		0.669		0.414		0.465

Q1 is set as the reference. ^a^ Odds ratios (95% confidence intervals). Models were adjusted for age, sex, education, marriage, smoking, BMI, total calories and total fat. ^b^ *p*-value for trends across the lowest quartile (Q1) to the highest quartile (Q4). * *p* < 0.05 for tested odds ratios. BMI = body mass index; DM = diabetes mellitus; CVD = cardiovascular disease; CHD = coronary heart disease; Q = quartile. MMP: monomethyl phthalate; MEP: monoethylphthalate; MnBP: mono-n-butylphthalate; MiBP: monoisobutylphthalate; MBzP: mono-benzylphthalate; MEHP: mono-2-ethylhexylphthalate; MEOHP: mono-2-ethyl-5-oxohexyphthalate; MEHHP: mono-2-ethyl-5-hydroxyhexylphthalate; MECPP: mono-2-ethyl-5-carboxypentylphthalate; MCMHP: mono-2-carboxymethyl-hexyl phthalate.

## Supplementary Materials

The following are available online at www.mdpi.com/1660-4601/14/6/598/s1, Table S1: The rotated eigenvectors of the three components after principal component analysis, Table S2: Logistic regression analyses of quartile metabolites of phthalates in association with self-reported DM and CVD (*n* = 2330).
