# Supplementary material for: Sex Differences in the Association of Urinary Concentrations of Phthalates Metabolites with Self-Reported Diabetes and Cardiovascular Diseases in Shanghai Adults
Source: Int J Environ Res Public Health. 2017 Jun 5;14(6):598. doi: 10.3390/ijerph14060598 (PMC5486284; doi:10.3390/ijerph14060598)
Supplement: Supplementary file 1 [file ijerph-14-00598-s001.pdf]

# Sex Differences in the Association of Urinary Concentrations of Phthalate Metabolites with Self-reported Diabetes and Cardiovascular Diseases in Shanghai Adults

**Ruihua Dong [1], Shanzhen Zhao [2], Han Zhang [1], Jingsi Chen [1], Meiru Zhang [1], Min Wang [2], Min Wu [1], Shuguang Li [1,*], Bo Chen [1,*]**

In order to assess the co-exposure, we performed principal component analysis (PCA) using the measured values of ten metabolites (Table S1). The major component accounted for 38.9% of the source variance and was dominated by the five metabolites of DEHP. We therefore assessed the co-exposure by calculating the micromolar sum of DEHP metabolites (ΣDEHP) including MEHP, MEHHP, MECPP, MEOHP and MCMHP.

**Table S1.** The rotated eigenvectors of the three components after principal component analysis.

| | **PC1** | **PC2** | **PC3** |
|---|---|---|---|
| MEOHP | 0.940 | 0.114 | 0.065 |
| MECPP | 0.920 | 0.146 | 0.040 |
| MCMHP | 0.886 | 0.098 | 0.075 |
| MEHHP | 0.846 | 0.140 | −0.065 |
| MEHP | 0.765 | 0.125 | −0.098 |
| MiBP | 0.135 | 0.809 | 0.041 |
| MnBP | 0.229 | 0.798 | 0.022 |
| MMP | 0.069 | 0.615 | −0.208 |
| MEP | 0.019 | 0.317 | 0.072 |
| MBzP | 0.006 | 0.026 | 0.976 |
| Eigenvalues | 3.894 | 1.850 | 1.029 |
| Variance explained (in %) | 0.389 | 0.185 | 0.103 |
| Cumulative variance (in %) | 0.389 | 0.574 | 0.677 |

Table S2 shows the adjusted (without adjusting the dietary factors) odds of each individual outcome (DM vs. normal; hypertension vs. normal; hyperlipidemia vs. normal; CHD vs. normal; stroke vs. normal; CVD vs. normal) by quartile of phthalate concentrations.

**Table S2.** Logistic regression analyses of quartile metabolites of phthalates in association with self-reported DM and CVD ($n$ = 2330).

| | | DM | Hypertension | Hyperlipidaemia | CHD | Stroke | CVD |
|---|---|---|---|---|---|---|---|
| MMP | Q2 vs. Q1 | 1.00 (0.61, 1.65) [a] | 0.97 (0.71, 1.33) | 1.33 (0.75, 2.34) | 1.27 (0.70, 2.30) | 2.25 (0.43, 11.81) | 1.36 (0.78, 2.39) |
| | Q3 vs. Q1 | 0.93 (0.57, 1.52) | 1.03 (0.76, 1.39) | 1.45 (0.84, 2.52) | 0.82 (0.43, 1.54) | 0.95 (0.13, 6.84) | 0.82 (0.45, 1.51) |
| | Q4 vs. Q1 | 1.55 (0.99, 2.42) | 1.23 (0.91, 1.66) | 2.06 (1.23, 3.44) * | 1.37 (0.79, 2.39) | 4.07 (0.82, 20.28) | 1.58 (0.94, 2.67) |
| | P for trend [b] | 0.060 | 0.159 | 0.005 | 0.457 | 0.131 | 0.213 |
| MEP | Q2 vs. Q1 | 1.04 (0.64, 1.68) | 1.04 (0.77, 1.41) | 0.70 (0.40, 1.22) | 1.20 (0.67, 2.16) | 1.02 (0.75, 1.41) | 1.43 (0.81, 2.53) |
| | Q3 vs. Q1 | 1.03 (0.64, 1.67) | 1.06 (0.79, 1.44) | 0.97 (0.58, 1.61) | 0.89 (0.48, 1.64) | 0.95 (0.69, 1.31) | 1.15 (0.65, 2.08) |
| | Q4 vs. Q1 | 1.47 (0.94, 2.30) | 1.15 (0.85, 1.56) | 1.42 (0.88, 2.27) | 1.39 (0.80, 2.43) | 1.28 (0.93, 1.75) | 1.18 (1.05, 3.10) |
| | P for trend | 0.096 | 0.354 | 0.035 | 0.379 | 0.007 | 0.061 |
| $M_iBP$ | Q2 vs. Q1 | 1.05 (0.67, 1.65) | 0.86 (0.64, 1.15) | 1.54 (0.93, 2.58) | 1.47 (0.84, 2.58) | 2.29 (0.44, 11.95) | 1.56 (0.91, 2.66) |
| | Q3 vs. Q1 | 1.16 (0.74, 1.84) | 0.81 (0.60, 1.09) | 1.27 (0.74, 2.15) | 0.94 (0.51, 1.75) | 0.58 (0.05, 6.49) | 0.93 (0.51, 1.70) |
| | Q4 vs. Q1 | 0.91 (0.57, 1.46) | 0.94 (0.69, 1.26) | 1.33 (0.79, 2.22) | 1.14 (0.63, 2.05) | 4.99 (1.03, 24.17) | 1.46 (0.84, 2.51) |
| | P for trend | 0.824 | 0.585 | 0.305 | 0.937 | 0.057 | 0.473 |
| $M_nBP$ | Q2 vs. Q1 | 0.73 (0.45, 1.19) | 0.80 (0.59, 1.08) | 1.41 (0.84, 2.38) | 0.61 (0.33, 1.11) | 1.58 (0.26, 9.64) | 0.67 (0.38, 1.18) |
| | Q3 vs. Q1 | 0.98 (0.62, 1.55) | 0.78 (0.58, 1.06) | 1.41 (0.842.36) | 0.83 (0.47, 1.47) | 2.86 (0.54, 15.22) | 0.94 (0.58, 1.67) |
| | Q4 vs. Q1 | 1.07 (0.69, 1.66) | 0.78 (0.58, 1.04) | 1.12 (0.66, 1.88) | 0.83 (0.48, 1.43) | 4.05 (0.79, 20.88) | 1.02 (0.61, 1.71) |
| | P for trend | 0.516 | 0.101 | 0.493 | 0.750 | 0.057 | 0.623 |
| $MB_zP$ | Q2 vs. Q1 | 0.66 (0.40, 1.07) | 0.76 (0.56, 1.03) | 0.89 (0.53, 1.51) | 0.61 (0.33, 1.14) | 1.69 (0.48, 5.92) | 0.75 (0.43, 1.31) |
| | Q3 vs. Q1 | 0.93 (0.60, 1.45) | 0.73 (0.54, 0.98) | 1.09 (0.67, 1.77) | 0.68 (0.38, 1.20) | 0.49 (0.09, 2.74) | 0.67 (0.38, 1.13) |
| | Q4 vs. Q1 | 1.05 (0.67, 1.63) | 0.98 (0.72, 1.31) | 1.21 (0.75, 1.98) | 1.22 (0.72, 2.05) | 0.76 (0.17, 3.47) | 1.15 (0.70, 1.89) |
| | P for trend | 0.529 | 0.809 | 0.321 | 0.427 | 0.397 | 0.680 |
| MEHP | Q2 vs. Q1 | 0.90 (0.56, 1.44) | 0.88 (0.65, 1.19) | 0.64 (0.38, 1.06) | 0.48 (0.25, 0.94) | 0.95 (0.25, 3.60) | 0.54 (0.30, 1.00) |
| | Q3 vs. Q1 | 0.92 (0.58, 1.46) | 0.71 (0.52, 0.96) | 0.52 (0.31, 0.88) | 0.87 (0.50, 1.53) | 0.67 (0.16, 2.89) | 0.84 (0.49, 1.42) |
| | Q4 vs. Q1 | 1.00 (0.64, 1.57) | 1.07 (0.79, 1.45) | 0.91 (0.58, 1.43) | 1.07 (0.63, 1.81) | 1.02 (0.26, 3.40) | 1.07 (0.65, 1.76) |
| | P for trend | 0.947 | 0.977 | 0.675 | 0.399 | 0.892 | 0.454 |
| MEOHP | Q2 vs. Q1 | 0.85 (0.50, 1.45) | 0.83 (0.62, 1.13) | 0.75 (0.43, 1.30) | 1.06 (0.60, 1.86) | 1.14 (0.28, 4.69) | 1.08 (0.64, 1.84) |
| | Q3 vs. Q1 | 1.69 (1.06, 2.70)* | 0.75 (0.55, 1.01) | 1.30 (0.79, 2.13) | 0.72 (0.40, 1.31) | 1.00 (0.22, 4.61) | 0.76 (0.44, 1.32) |
| | Q4 vs. Q1 | 1.52 (0.94, 2.45) | 1.02 (0.75, 1.38) | 1.25 (0.76, 2.07) | 0.96 (0.54, 1.70) | 1.69 (0.44, 6.56) | 1.04 (0.61, 1.78) |
| | P for trend | 0.011 | 0.942 | 0.124 | 0.599 | 0.493 | 0.802 |
| MEHHP | Q2 vs. Q1 | 1.72 (0.86, 2.36) | 0.67 (0.51, 0.95) | 0.80 (0.47, 1.37) | 0.57 (0.32, 1.03) | 2.16 (0.38, 12.15) | 0.68 (0.39, 1.18) |
| | Q3 vs. Q1 | 1.40 (0.84, 2.32) | 0.79 (0.58, 1.07) | 1.31 (0.81, 2.14) | 0.69 (0.39, 1.21) | 3.52 (0.68, 18.16) | 0.85 (0.50, 1.44) |
| | Q4 vs. Q1 | 1.70 (1.03, 2.79) | 1.08 (0.79, 1.47) | 0.85 (0.50, 1.46) | 0.72 (0.41, 1.28) | 2.48 (0.44, 14.08) | 0.84 (0.49, 1.44) |
| | P for trend | 0.059 | 0.387 | 0.884 | 0.443 | 0.246 | 0.771 |
| MECPP | Q2 vs. Q1 | 1.29 (0.76, 2.17) | 0.87 (0.64, 1.17) | 0.83 (0.49, 1.43) | 0.75 (0.41, 1.36) | 1.00 (0.25, 4.11) | 0.77 (0.44, 1.35) |
| | Q3 vs. Q1 | 1.77 (1.08, 2.90) * | 0.88 (0.66, 1.19) | 1.41 (0.87, 2.29) | 0.96 (0.55, 1.68) | 1.10 (0.27, 4.54) | 0.97 (0.58, 1.64) |
| | Q4 vs. Q1 | 1.86 (1.151, 3.12) * | 1.13 (0.83, 1.54) | 1.20 (0.72, 2.01) | 0.98 (0.55, 1.74) | 1.34 (0.32, 5.61) | 1.01 (0.59, 1.74) |
| | P for trend | 0.005 | 0.455 | 0.175 | 0.811 | 0.684 | 0.741 |

**Table S2.** *Cont*.

| | | | | | | | |
|---|---|---|---|---|---|---|---|
| MCMHP | Q2 vs. Q1 | 1.17 (0.72, 1.90) | 0.98 (0.72, 1.31) | 1.37 (0.80.2.34) | 1.06 (0.61, 1.83) | 1.31 (0.34, 4.96) | 1.10 (0.66, 1.83) |
| | Q3 vs. Q1 | 1.55 (0.98, 2.46) | 0.82 (0.61, 1.11) | 1.96 (1.18, 3.25) * | 0.65 (0.36, 1.17) | 0.89 (0.20, 4.07) | 0.66 (0.38, 1.16) |
| | Q4 vs. Q1 | 1.25 (0.77, 2.03) | 1.17 (0.86, 1.59) | 1.55 (0.90, 2.65) | 1.02 (0.59, 1.78) | 1.25 (0.30, 5.17) | 1.04 (0.62, 1.75) |
| | P for trend | 0.207 | 0.577 | 0.047 | 0.661 | 0.676 | 0.895 |
| ΣDEHP | Q2 vs. Q1 | 0.98 (0.59, 1.63) | 0.78 (0.57, 1.06) | 0.91 (0.54, 1.53) | 0.71 (0.39, 1.29) | 1.10 (0.31, 3.93) | 0.76 (0.44, 1.32) |
| | Q3 vs. Q1 | 1.19 (0.74, 1.92) | 0.82 (0.60, 1.11) | 0.92 (0.55, 1.52) | 0.62 (0.35, 1.12) | 0.42 (0.08, 2.24) | 0.59 (0.34, 1.03) |
| | Q4 vs. Q1 | 1.42 (0.89, 2.27) | 1.12 (0.83, 1.51) | 1.16 (0.71, 1.89) | 0.99 (0.57, 1.70) | 0.97 (0.25, 3.80) | 0.98 (0.59, 1.64) |
| | *p* for trend | 0.088 | 0.410 | 0.520 | 0.965 | 0.683 | 0.827 |

Q1 is set as the reference. [a] Odds ratios (95% confidence intervals). Models were adjusted for age, sex, education, marriage, smoking, BMI. [b] *p*-value for trends across the lowest quartile (Q1) to the highest quartile (Q4). * $p < 0.05$ for tested odds ratios. BMI = body mass index; DM = diabetes mellitus; CVD = cardiovascular disease; CHD = coronary heart disease; Q = quartile.
